# Quaternary Radiation of Spring Ephemerals

**DOI:** 10.1002/pei3.70021

**Published:** 2025-01-24

**Authors:** Soichi Osozawa, Cunio Nackejima

**Affiliations:** ^1^ Institute of Geology and Paleontology, Faculty of Science Tohoku University Sendai Japan; ^2^ Department of Tourism Industry Meo University Nago Japan

**Keywords:** adaptive radiation, *Asarum*, BEAST v1.10.4, climatic change, PopART version 1.7, spring ephemerals, vicariance, *Viola*

## Abstract

The emergence of East Asian spring ephemerals and the unique ecosystem can be attributed primarily to vicariance, brought about by the Quaternary rifting of the Okinawa Trough, the formation of the East China Sea, and the isolation of the island chains of Ryukyu, Japan, and Taiwan from the Asian continent. The northern forests of Japan, dominated by *Fagus crenata* and the associated *Quercus crispula*, present a captivating display of spring‐flowering ephemerals, including *Erythronium japonicum*, *Viola bissetii*, *Anemone pseudoaltaica*, and *Corydalis fukuharae*. Among these, *Asarum* is also considered part of the spring ephemerals. Our primary focus for phylogenetic analyses, which included constructing a haplotype network using PopART version 1.7 and molecular dating with BEAST v1.10.4, was on the genus *Asarum*. In the BEAST analysis, we set the Quaternary geological event calibration at 1.55 ± 0.15 Ma and applied pre‐Quaternary fossil calibrations. When we input 1.55 Ma into BEAST, the analysis suggested that spring ephemerals underwent a simultaneous splitting and diversification event around that time, approximately 1.55 Ma, during the Quaternary period. The differentiation of these species is more likely a result of adaptive radiation rather than vicariance, particularly given their partially sympatric distribution and occurrence across islands. This radiation likely originated from the most recent common ancestor of the ingroup species of spring ephemerals and can be seen as a response to significant environmental changes associated with the formation of the East China Sea around 1.55 Ma. Notably, species such as *Asarum megacalyx* and *Fagus crenata* exhibit adaptations to heavy snowfall, further supporting this idea. The spring ephemerals in beech or oak forests in North America and Europe may have radiated and diversified as a result of Quaternary global climatic changes.

## Graphical abstract

The emergence of spring ephemerals can be primarily attributed to vicariance, with secondary adaptation to Quaternary climatic changes.

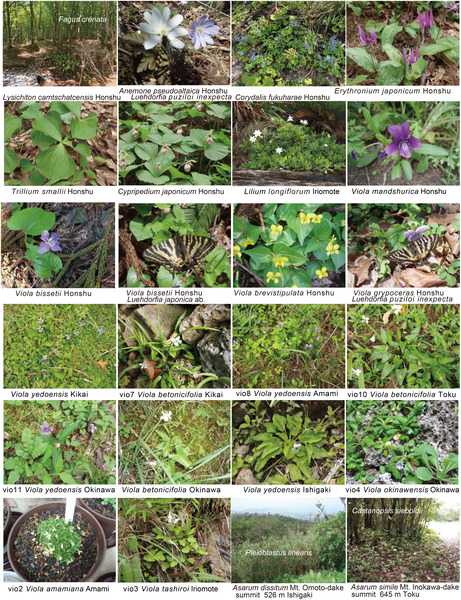

## 1 Introduction

**FIGURE 1:**
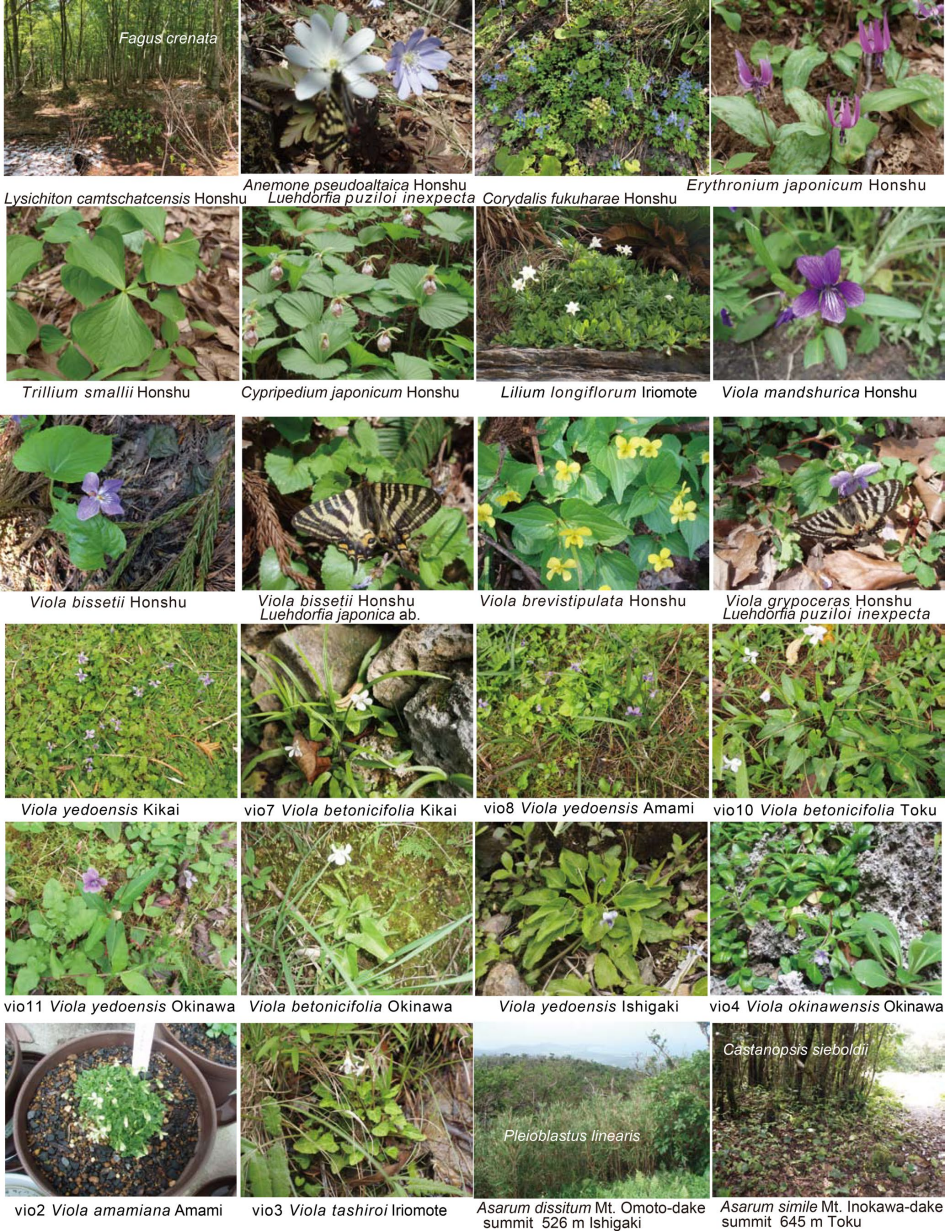
Field photos of spring ephemerals. *Viola amamiana* was cultivated by transplanting from the wild.

**FIGURE 2:**
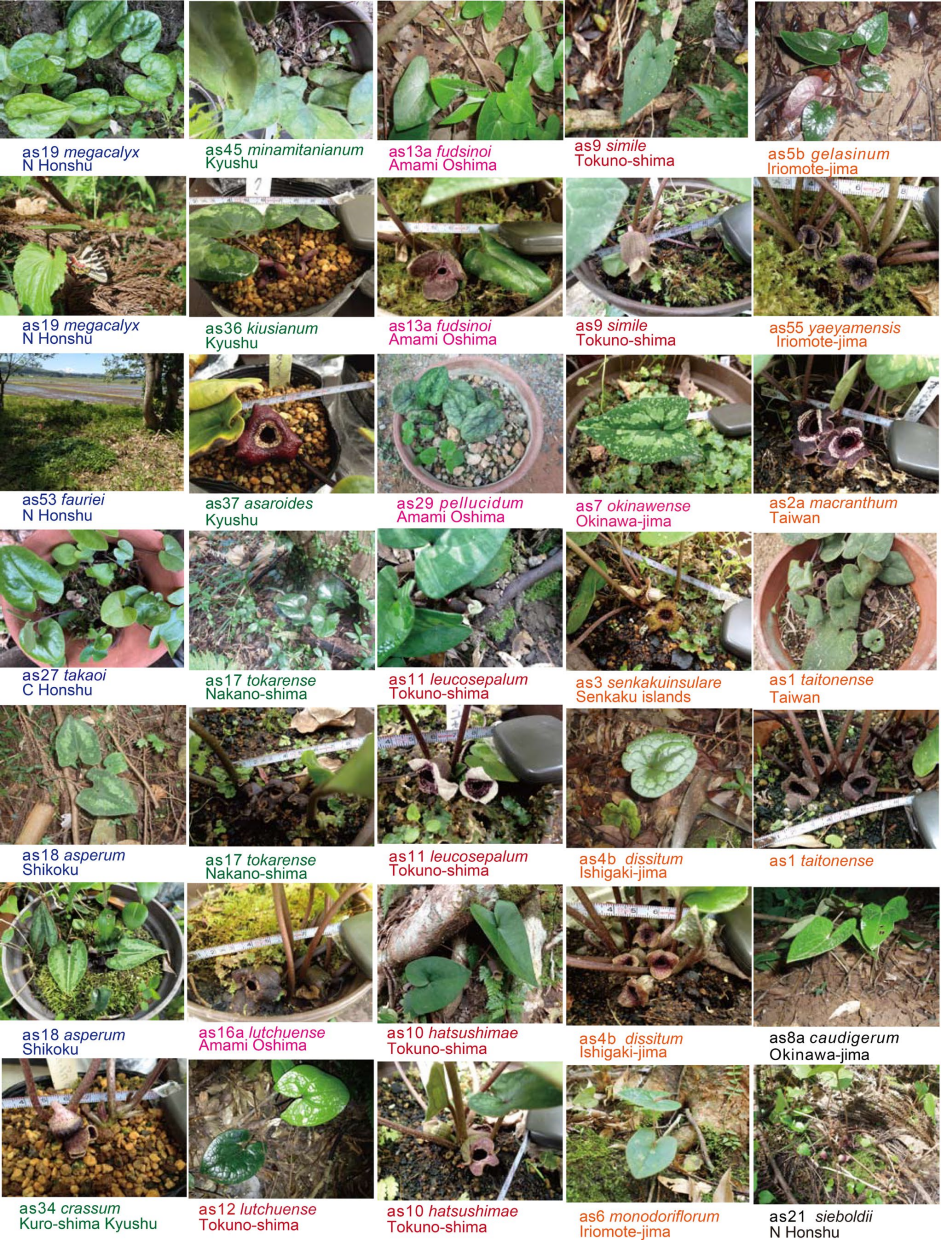
*Asarum* (wild ginger) in their natural habitat, flowering where possible.

Forest herbs contribute significantly to the species diversity of vascular plants in temperate deciduous forests characterized by beech or oak trees in North America, Europe, and East Asia. Their seasonal dormancy is an adaptive strategy that allows herbaceous plants to optimize their fitness by assimilating carbon during favorable environmental periods (Augspurger and Salk 2017). One such forest can be found in northeast Japan, with the beech forest along the Japan Sea side being the most representative. The herb layer in these forests consists of perennial wildflowers that emerge rapidly under the early spring sunshine and then recede to their underground parts in the shaded canopy of mature leaves. These flowering plants are commonly referred to as spring ephemerals, and some examples found in Japanese beech forests include *Erythronium japonicum*, *Trillium smallii*, *Viola bissetii*, *Viola brevistipulata*, *Anemone pseudoaltaica*, *Corydalis fukuharae*, and *Adonis amurensis* (Figure 1). In addition to these modest bloomers that maintain their green foliage throughout the year, the *Heterotropa* section of *Asarum*, the primary focus of our study (Figure 2), is also considered a spring ephemeral. The *Luehdorfia* butterfly, a notable spring ephemeral or “primavera,” feeds on *Heterotropa* (Figure 1) and will be discussed in a separate paper.

The flowering of spring ephemerals, along with shrubs such as *Cerasus sargentii*, *Rhododendron albrechtii*, and *Camellia rusticana*, coincides with the melting of snow, synchronized with the sprouting and leafing of the tall *Fagus crenata* forest. The pale green foliage gradually spreads from lower to higher elevations, dramatically transforming the landscape from winter to spring as the days progress. The origin of spring ephemerals may be linked to the current snowy climate along the Japan Sea coastal area, where the adaptation helps plants avoid the harshness of winter cold, heavy snowfall, and late‐spring canopy shade (Augspurger and Salk 2017), which are all influenced by climatic changes (Petrauski et al. 2019; Kudo and Cooper 2019; Gao et al. 2022; Wang, Zhu, et al. 2023; Wang, Liu, et al. 2023; Xiao et al. 2024).

**FIGURE 3:**
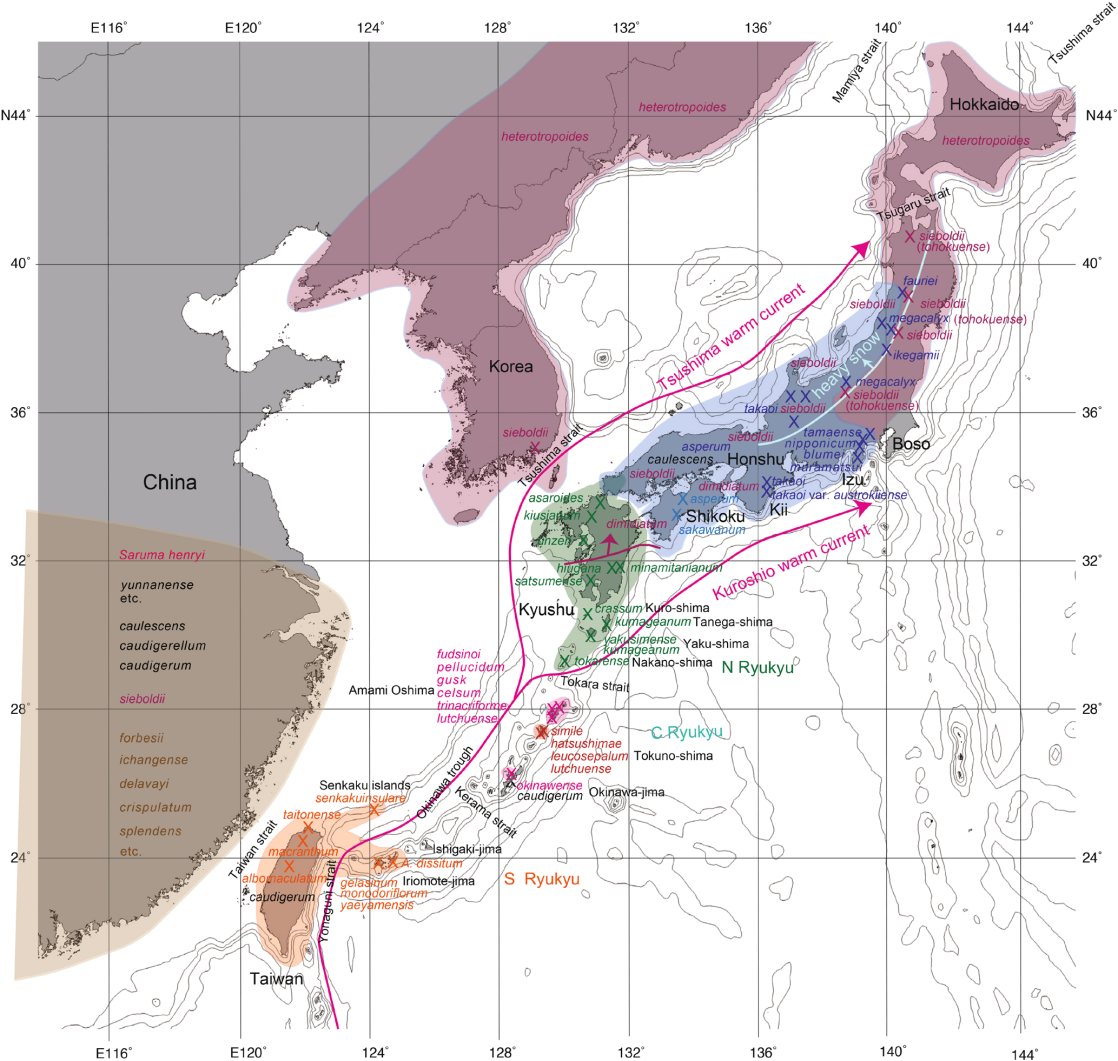
Distribution map of *Asarum* wild gingers in the Japan–Ryukyu–Taiwan islands and China.

**FIGURE 4:**
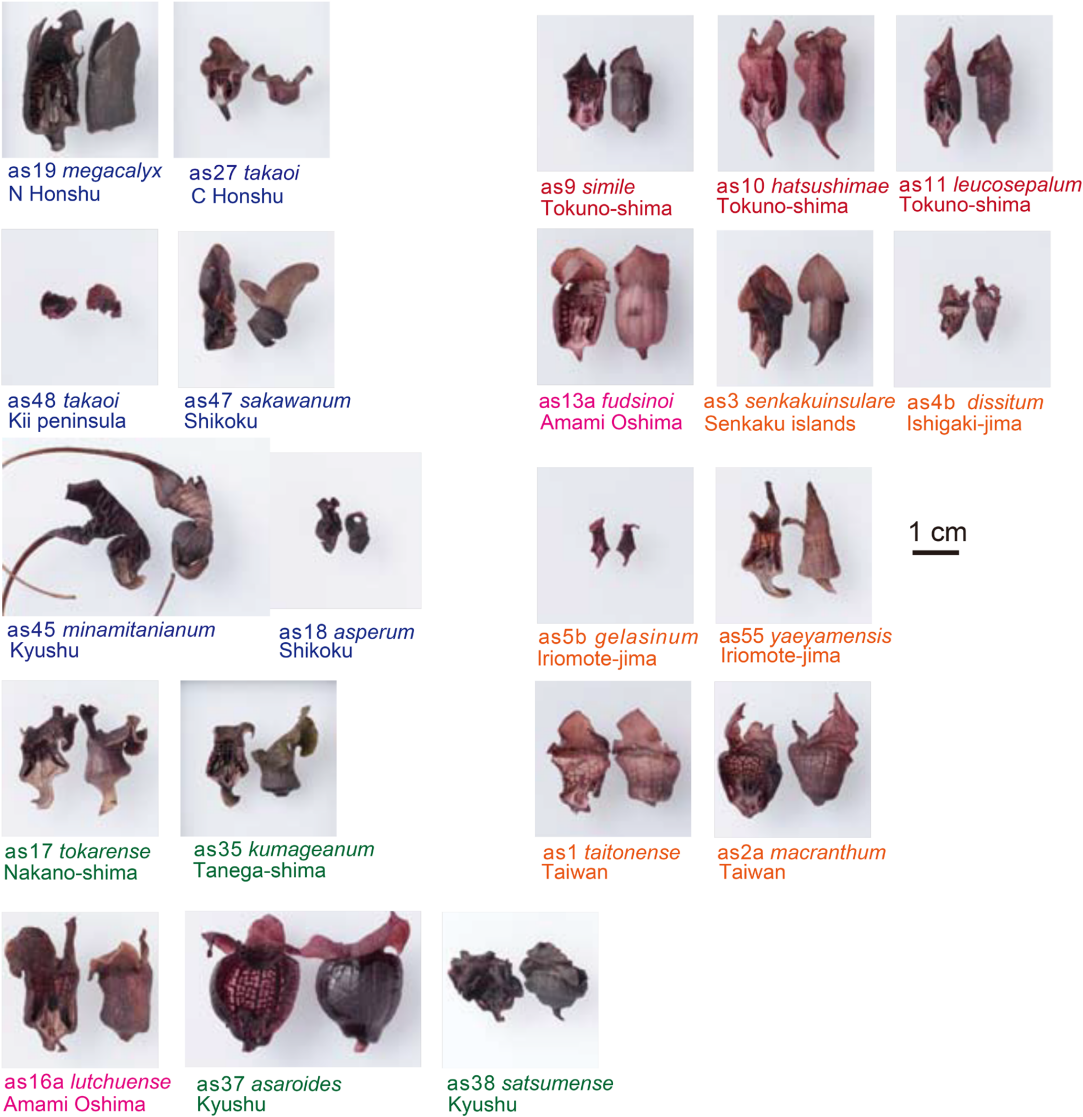
Calyx variation in *Asarum* wild gingers.

The Tsushima warm current began to flow into the Japan Sea around 1.55 million years ago (Ma) during the Quaternary period based on our field geological survey (Osozawa et al. 2012, c.f., Nemoto 2006), leading to a significant climate change in the Japan Sea area. During winter, the northwestern seasonal wind crossing the Japan Sea absorbs moisture from the warm water, resulting in heavy snowfall along the coastal areas on the western side of the northern Honshu Mountain range (Figure 3). Just like *Fagus crenata* and *Camellia rusticana*, *Asarum megacalyx* (*Heterotropa*) may have adapted to this snowy environment and subsequently experienced diversification in this region. The concept of adaptation to a snowy climate, including the hypothesis that the increased size of the calyx (Figure 4) helps prevent heavy snow accumulation, was originally proposed by Hiura (1978). However, the exact geological timing of the opening of the Tsushima strait and the inflow of the Tsushima current into the Japan Sea (refer to Figure 3) was uncertain in 1978.

The formation of the Okinawa Trough, along with other major straits such as the Tsushima and Taiwan Straits (Figure 3), led to the simultaneous separation and isolation of the islands of Japan, Ryukyu, and Taiwan from the Asian continent. These seaways likely acted as significant barriers, triggering vicariance events. The geologically robust geological event calibration age of 1.55 Ma as the time of most recent common ancestor (tMRCA) should be applied to current phylogenetic analyses (Osozawa 2023; Osozawa and Nel 2024) when studying the species diversity of *Heterotropa*, which extends not only in Japan but also includes the Ryukyu Islands, Taiwan, and China (Figure 3). The initial diversification of *Heterotropa* is thought to have been caused by allopatric range fragmentation in the island arcs stretching from Taiwan to mainland Japan via the Ryukyu Islands (Takahashi and Setoguchi 2017). Additionally, *Viola* (Marcussen et al. 2022) and *Fagus* (Jiang et al. 2021) have also undergone similar diversification in East Asia.

To better understand the origin, factors, and interrelationships of spring ephemerals primarily on the islands of East Asia, we are constructing a new haplotype network and dated phylogeny for these plants. We are also conducting comparative analyses in light of the significant environmental changes that have occurred in the region (Osozawa et al. 2012). This research aims to shed light on the evolutionary history of the East Asian deciduous forest ecosystem, including its spring ephemerals. Additionally, our findings are relevant to North American and European forests as spring ephemerals from these regions were also partially included in our analyses.

## 2 Materials and Methods

The present study did not involve endangered or protected species. Collection in the Ryukyu Islands was before the designation of National Park in 2016. No specific permission was required outside the national parks and private areas.

### 2.1 *Asarum* Sampling: Back Ground

The genus *Asarum* encompasses a total of 128 described species (Okuyama et al. 2020), with the majority being found in East Asia. Approximately 50 species are known from the Japan and Ryukyu Islands (Sugawara 2006; Matsuda et al. 2017; Takahashi and Setoguchi 2017), while 15 species are reported from North America as part of the section Hexastylis (Kelly 1998; Sinn, Kelly, and Freudenstein 2015a, 2015b). Our study primarily focuses on the genus *Asarum* from the Japan and Ryukyu Islands, while also including Taiwan and China.

**FIGURE 5:**
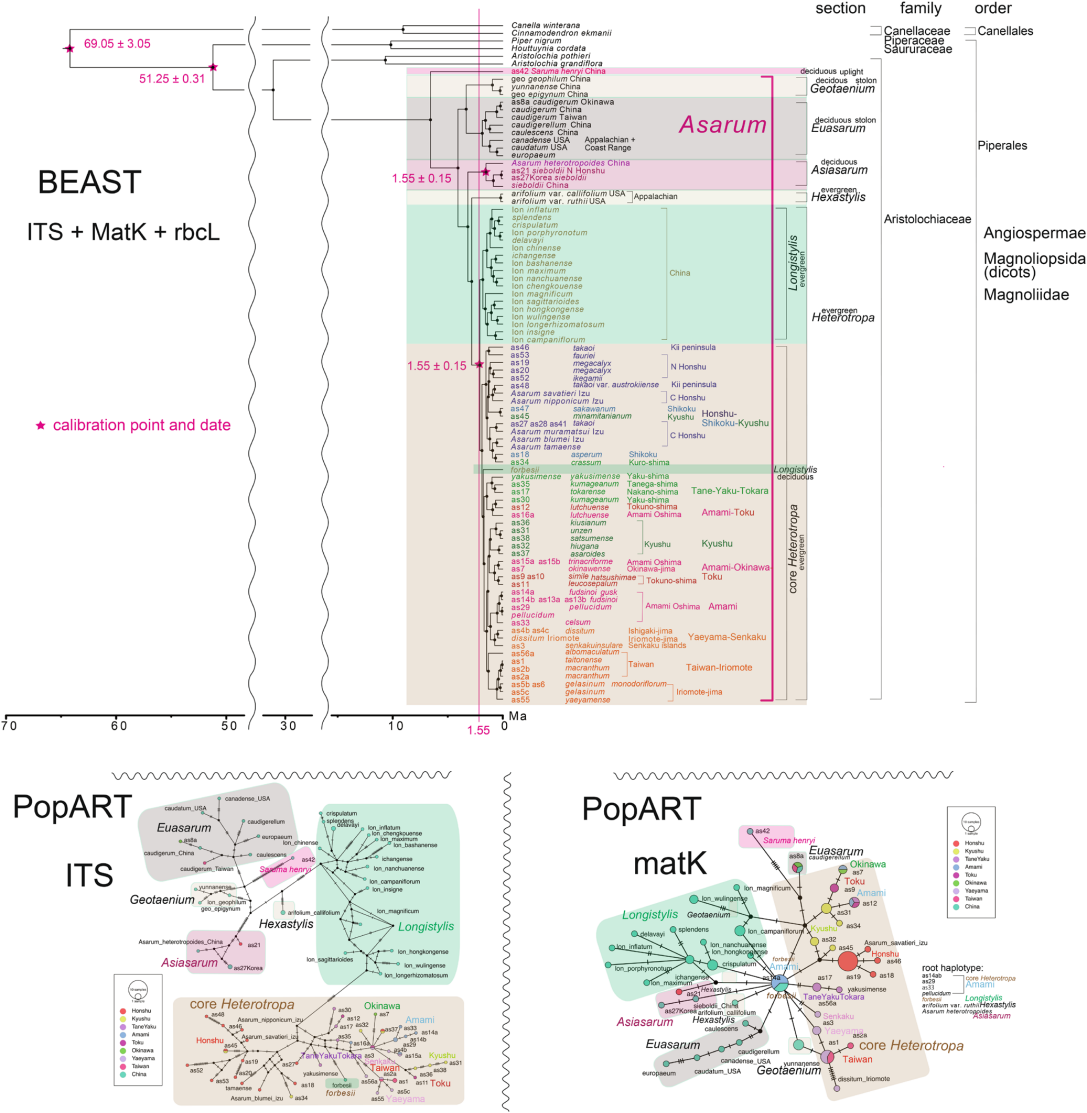
Top: Bayesian inference tree of *Asarum* (Piperales; with Canellales included) based on a combined sequence of ITS (694 bp), matK (833 bp), and rbcL (544 bp), constructed using BEAST v1.10.4. Bottom: ITS and matK haplotype network of *Asarum* generated using PopART.

In addition to the section Hexastylis (evergreen), the genus *Asarum* consists of four other sections (Figure 5): *Geotaenium* (deciduous, stolon; southern China and Taiwan; Takahashi and Setoguchi 2017), *Euasarum* (deciduous, stolon; China, Taiwan, Okinawa, North America, and Europe; 16 species in Kelly 1998), *Asiasarum* (deciduous; 7 species in Japan; Yamaji, Nakamura, et al. 2007), and *Heterotropa* (evergreen and extensively diversified; our main focus). Previous studies have explored the phylogeny of these sections, particularly section *Hexastylis* and *Euasarum*, conducted by Kelly (1998), Sugawara et al. (2005), and Sinn, Kelly, and Freudenstein (2015a, 2015b). Section *Asiasarum* is found in southern China, Korea, Primorsky, Hokkaido, Honshu, Shikoku, and northern Kyushu, but not in the Ryukyu and Taiwan islands or northern China (Figure 3), and has also been extensively studied, with species subdivisions proposed by Yamaji, Fukuda, et al. (2007).

The majority of *Asarum* species belong to the section *Heterotropa*, which is distributed in the Ryukyu Islands, as well as the Japan and Taiwan islands, and southern China (Figure 3). However, they are not known to occur in northern China, Korea, Primorsky, Hokkaido, and northern Honshu. Our primary focus is on the insular *Heterotropa*, which encompasses 46 endemic species in our analyses (Table 1), out of the total of 72 species found in East Asia (Matsuda et al. 2017). Until recent studies by Matsuda et al. (2017), Takahashi and Setoguchi (2017), and Okuyama et al. (2020), *Heterotropa* species have not been extensively explored and systematically studied. We also include *Heterotropa* species from southern China (referred to as Longistylis by Sinn, Kelly, and Freudenstein 2015b; Figure 5) for comparison, as Chinese sequence data have become available in GenBank/DDJB.

**TABLE 1:** *Asarum* and *Viola* species analyzed in this study, along with related data uploaded to GenBank/DDBJ.

Isolate	Country	*Asarum* species	Section	Calyx color	Calyx shape	Calyx size	Leaf	Stem	Accession number ITS	Accession number matK	Accession number rbcL	Collected by
as53	Japan: Honshu, Yamagata, Sakekawa	*A. fauriei*	*Heterotropa*	Light purplish	Cylindrical	Moderate	Evergreen	Erect	LC669873	LC670174	LC670219	Soichi Osozawa
as19	Japan: Honshu, Yamagata, Nishikawa	*A. megacalyx*	Heterotropa	Purplish	Cylindrical	Giant	Evergreen	Erect	LC068456	LC068502	LC068548	Soichi Osozawa
as20	Japan: Honshu, Yamagata, Tsuruoka	*A. megacalyx*	Heterotropa	Purplish	Cylindrical	Giant	Evergreen	Erect	LC068457	LC068503	LC068549	Soichi Osozawa
as52	Japan: Honshu, Fukushima, Mishima	*A. ikegamii*	*Heterotropa*	Light purplish	Cylindrical	Moderate	Evergreen	Erect	LC669872	LC670173	LC670218	Iichi Tsunoda
as27	Japan: Honshu, Toyama, Osawano	*A. fauriei*	*Heterotropa*	Light purplish	Cylindrical	Small	Evergreen	Erect	LC068464	LC068510	LC068556	Kyoji Osozawa
as28	Japan: Honshu, Ishikawa, Tsurugi	*A. fauriei*	*Heterotropa*	Light purplish	Cylindrical	Small	Evergreen	Erect	LC068465	LC068511	LC068557	Kyoji Osozawa
as41	Japan: Honshu, Gifu, Yamagata	*A. fauriei*	*Heterotropa*	Light purplish	Cylindrical	Small	Evergreen	Erect	LC068473	LC068519	LC068565	Akio Komano
as46	Japan: Honshu, Kii peninsula	*A. takaoi*	*Heterotropa*	Light purplish	Cylindrical	Moderate	Evergreen	Erect	LC669869	LC670170	LC670215	Kinichi Watanabe
as48	Japan: Honshu, Kii peninsula	*A. takaoi var. austrokiiense*	*Heterotropa*	Light purplish	Cylindrical	Moderate	Evergreen	Erect	LC669871	LC670172	LC670217	Kinichi Watanabe
as18	Japan: Shikoku, Ehime, ShikokuChuo	*A. asperum*	Heterotropa	Light purplish	Cylindrical	Moderate	Evergreen	Erect	LC068455	LC068501	LC068547	Soichi Osozawa
as47	Japan: Shikoku, Kochi	*A. sakawanum*	*Heterotropa*	Light purplish	Cylindrical	Moderate	Evergreen	Erect	LC669870	LC670171	LC670216	Kinichi Watanabe
as45	Japan: Kyushu, Miyazaki	*A. minamitanianum*	*Heterotropa*	Light purplish	Stringy	Moderate	Evergreen	Erect	LC669868	LC670169	LC670214	Kinichi Watanabe
as34	Japan: Kyushu, Miyazaki	*A. crassum*	*Heterotropa*	Light purplish	Cylindrical	Moderate	Evergreen	Erect	LC669866	LC670167	LC670212	Kinichi Watanabe
as36	Japan: Kyushu, Fukuoka	*A. kiusianum*	*Heterotropa*	Purplish	Vase	Moderate	Evergreen	Erect	LC068470	LC068516	LC068562	Kinichi Watanabe
as37	Japan: Kyushu, Fukuoka	*A. asaroides*	*Heterotropa*	Purplish	Vase	Giant	Evergreen	Erect	LC068471	LC068517	LC068563	Kinichi Watanabe
as31	Japan: Kyushu, Nagasaki	*A. unzen*	*Heterotropa*	Purplish	Vase	Moderate	Evergreen	Erect	LC068467	LC068513	LC068559	Kinichi Watanabe
as32	Japan: Kyushu, Miyazaki	*A. hiugana*	*Heterotropa*	Purplish	Vase	Moderate	Evergreen	Erect	LC068468	LC068514	LC068560	Kinichi Watanabe
as38	Japan: Kyushu, Kagoshima	*A. satsumense*	*Heterotropa*	Purplish	Vase	Moderate	Evergreen	Erect	LC068472	LC068518	LC068564	Kinichi Watanabe
as30	Japan: Kyushu, Yaku‐shima	*A. kumageanum*	*Heterotropa*	Purplish	Vase	Moderate	Evergreen	Erect	LC068466	LC068512	LC068558	Kinichi Watanabe
as35	Japan: Kyushu, Tanega‐shima	*A. kumageanum*	*Heterotropa*	Purplish	Vase	Moderate	Evergreen	Erect	LC068469	LC068515	LC068561	Kinichi Watanabe
as17	Japan: Ryukyu, Tokara, Nakano‐shjima; Mt. O‐take; 979 m	*A. tokarense*	*Heterotropa*	Purplish	Vase	Moderate	Evergreen	Erect	LC068454	LC068500	LC068546	Soichi Osozawa
as16a	Japan: Ryukyu, Amami Oshima; Tatsugo; at 200 m altitude	*A. lutchuense*	Heterotropa	Purplish	Vase	Moderate	Evergreen	Erect	LC068452	LC068498	LC068544	Soichi Osozawa
as16b	Japan: Ryukyu, Amami Oshima; Tatsugo; at 200 m altitude	*A. lutchuense*	Heterotropa	Purplish	Vase	Moderate	Evergreen	Erect	LC068453	LC068499	LC068545	Soichi Osozawa
as13a	Japan: Ryukyu, Amami Oshima; Mt. Yuwan‐dake; 694 m	*A. fudsinoi*	Heterotropa	Light purplish	Cylindrical	Moderate	Evergreen	Erect	LC068446	LC068492	LC068538	Soichi Osozawa
as13b	Japan: Ryukyu, Amami Oshima; Mt. Yuwan‐dake; 694 m	*A. fudsinoi*	Heterotropa	Light purplish	Cylindrical	Moderate	Evergreen	Erect	LC068447	LC068493	LC068539	Soichi Osozawa
as14a	Japan: Ryukyu, Amami Oshima; Mt. Yuwan‐dake; 694 m	*A. fudsinoi*	Heterotropa	Light purplish	Cylindrical	Moderate	Evergreen	Erect	LC068448	LC068494	LC068540	Soichi Osozawa
as14b	Japan: Ryukyu, Amami Oshima; Mt. Yuwan‐dake; 694 m	*A. fudsinoi*	Heterotropa	Light purplish	Cylindrical	Moderate	Evergreen	Erect	LC068449	LC068495	LC068541	Soichi Osozawa
as33	Japan: Ryukyu, Amami Oshima; Mt. Yuwan‐dake; 694 m	*A. celsum*	Heterotropa	Light purplish	Cylindrical	Small	Evergreen	Erect	LC669865	LC670166	LC670211	Kinichi Watanabe
as15a	Japan: Ryukyu, Amami Oshima, Uke‐jima; Mt. O‐dake	*A. trinacriforme*	Heterotropa	Light purplish	Cylindrical	Moderate	Evergreen	Erect	LC068450	LC068496	LC068542	Cunio Nackejima
as15b	Japan: Ryukyu, Amami Oshima, Uke‐jima; Mt. O‐dake	*A. trinacriforme*	Heterotropa	Light purplish	Cylindrical	Moderate	Evergreen	Erect	LC068451	LC068497	LC068543	Cunio Nackejima
as9	Japan: Ryukyu, Tokuno‐shima; Mt. Inokawa‐dake; 645 m	*A. simile*	Heterotropa	Light purplish	Cylindrical	Moderate	Evergreen	Erect	LC068442	LC068488	LC068534	Soichi Osozawa
as10	Japan: Ryukyu, Tokuno‐shima; Mt. Amagi‐dake; 533 m	*A. hatsushimae*	Heterotropa	Light purplish	Cylindrical	Moderate	Evergreen	Erect	LC068443	LC068489	LC068535	Soichi Osozawa
as11	Japan: Ryukyu, Tokuno‐shima; Mt. Sasontsuji‐dake; 496 m	*A. leucosepalum*	Heterotropa	Purplish	Vase	Moderate	Evergreen	Erect	LC068444	LC068490	LC068536	Soichi Osozawa
as12	Japan: Ryukyu, Tokuno‐shima; Mt. Sasontsuji‐dake; at 400 m altitude	*A. lutchuense*	Heterotropa	Light purplish	Cylindrical	Moderate	Evergreen	Erect	LC068445	LC068491	LC068537	Soichi Osozawa
as7	Japan: Ryukyu, Okinawa‐jima, Motobu; Mt. Katsuu‐dake; 452 m	*A. okinawense*	Heterotropa	Light purplish	Cylindrical	Small	Evergreen	Erect	LC068438	LC068484	LC068530	Cunio Nackejima
as3	Japan: Ryukyu, Senkaku; Uotsuri‐jima	*A. senkakuinsulare*	Heterotropa	Light purplish	Cylindrical	Moderate	Evergreen	Erect	LC068432	LC068478	LC068524	Kinichi Watanabe
as4b	Japan: Ryukyu, Ishigaki‐jima; Mt. Omoto‐dake; 526 m	*A. dissitum*	Heterotropa	Light purplish	Cylindrical	Moderate	Evergreen	Erect	LC068433	LC068479	LC068525	Soichi Osozawa
as4c	Japan: Ryukyu, Ishigaki‐jima; Mt. Omoto‐dake; 526 m	*A. dissitum*	Heterotropa	Light purplish	Cylindrical	Moderate	Evergreen	Erect	LC068434	LC068480	LC068526	Soichi Osozawa
as5b	Japan: Iriomote‐jima; Mt. Koza‐dake; 420 m	*A. gelasinum*	Heterotropa	Purplish	Vase	Moderate	Evergreen	Erect	LC068435	LC068481	LC068527	Soichi Osozawa
as5c	Japan: Iriomote‐jima; Mt. Koza‐dake; 420 m	*A. gelasinum*	Heterotropa	Purplish	Vase	Moderate	Evergreen	Erect	LC068436	LC068482	LC068528	Soichi Osozawa
as6	Japan: Iriomote‐jima; Kanpira waterfall; 100 m	*A. monodoriflorum*	Heterotropa	Purplish	Vase	Moderate	Evergreen	Erect	LC068437	LC068483	LC068529	Soichi Osozawa
as55	Japan: Iriomote‐jima	*A. yaeyamense*	Heterotropa	Purplish	Vase	Moderate	Evergreen	Erect	LC669874	LC670175	LC670220	Kinichi Watanabe
as1	Taiwan: Yangmingshan; Mt. Datun‐shan; 1087 m	*A. taitonense*	Heterotropa	Purplish	Vase	Moderate	Evergreen	Erect	LC068429	LC068475	LC068521	Kyoji Osozawa
as2a	Taiwan: Wusubi; at 500 m altitude	*A. macranthum*	Heterotropa	Purplish	Vase	Moderate	Evergreen	Erect	LC068430	LC068476	LC068522	Soichi Osozawa
as2b	Taiwan: Wusubi; at 500 m altitude	*A. macranthum*	Heterotropa	Purplish	Vase	Moderate	Evergreen	Erect	LC068431	LC068477	LC068523	Soichi Osozawa
as56a	Taiwan: Alishan; at 900 m altitude	*A. albomaculatus*	Heterotropa	Purplish	Vase	Moderate	Evergreen	Erect	LC669875	LC670176	LC670221	Soichi Osozawa
as8a	Japan: Ryukyu, Okinawa‐jima, Motobu; Mt. Yae‐dake; 453 m	*A. caudigerum*	Euasarum	Purplish	Stringy	Moderate	deciduous	Stolon	LC068439	LC068485	LC068531	Cunio Nackejima
as8b	Japan: Ryukyu, Okinawa‐jima, Motobu; Mt. Yae‐dake; 453 m	*A. caudigerum*	Euasarum	Purplish	Stringy	Moderate	deciduous	Stolon	LC068440	LC068486	LC068532	Cunio Nackejima
as8c	Japan: Ryukyu, Okinawa‐jima, Motobu; Mt. Yae‐dake; 453 m	*A. caudigerum*	Euasarum	Purplish	Stringy	Moderate	deciduous	Stolon	LC068441	LC068487	LC068533	Cunio Nackejima
as21	Japan: Honshu, Yamagata, Tsuruoka	*A. sieboldii*	Asiasarum	Purplish	Bell	Moderate	deciduous	Erect	LC068458	LC068504	LC068550	Soichi Osozawa
as22	Japan: Honshu, Aomori, Hirosaki	*A. sieboldii*	Asiasarum	Purplish	Bell	Moderate	deciduous	Erect	LC068459	LC068505	LC068551	Soichi Osozawa
as23	Japan: Honshu, Miyagi, Sendai	*A. sieboldii*	Asiasarum	Purplish	Bell	Moderate	deciduous	Erect	LC068460	LC068506	LC068552	Soichi Osozawa
as25	Japan: Honshu, Yamagata, Tozawa	*A. sieboldii*	Asiasarum	Purplish	Bell	Moderate	deciduous	Erect	LC068461	LC068507	LC068553	Soichi Osozawa
as26	Japan: Honshu, Nagano, Sakae	*A. sieboldii*	Asiasarum	Purplish	Bell	Moderate	deciduous	Erect	LC068462	LC068508	LC068554	Kanjiro Ogura
as27Korea	Korea: Busan	*A. sieboldii*	Asiasarum	Purplish	Bell	Moderate	deciduous	Erect	LC068463	LC068509	LC068555	Soichi Osozawa
as42	China	*Saruma henryi*		Yellow	Petal	Moderate	deciduous	Erect	LC068474	LC068520	LC068566	Akio Komano

isolate	Country	Species							Accession number ITS	Accession number matK	Accession number rbcL	Collected by
mono1	Japan: Honshu, Fukushima, Mishima	*Iris ensata*							LC669876	LC670177	LC670222	Soichi Osozawa
mono3	Japan: Honshu, Miyagi, Sendai (unknown origin)	*Convallaria majalis*							LC669877	LC670178	LC670223	Soichi Osozawa
mono6	Japan: Ryukyu, Okinawa‐jima, Motobu; Mt. Katsuu‐dake; 452 m	*Arisaema ringens*							LC669878	LC670179	LC670224	Soichi Osozawa
dic20	Japan: Honshu, Miyagi, Sendai (unknown origin)	*Magnolia liliiflora*							LC669879	LC670180	LC670225	Soichi Osozawa
dic23	Japan: Honshu, Miyagi, Sendai (unknown origin)	*Buddleja davidii*							LC669880	LC670181	LC670226	Soichi Osozawa
dic32	Japan: Honshu, Miyagi, Sendai (unknown origin)	*Prunus mume*							LC669881	LC670182	LC670227	Soichi Osozawa
dic34	Japan: Honshu, Miyagi, Sendai (unknown origin)	*Euonymus alatus*							LC669882	LC670183	LC670228	Soichi Osozawa
dic35	Japan: Honshu, Miyagi, Sendai, Tohoku University	*Chloranthus japonicus*							LC669883	LC670184	LC670229	Soichi Osozawa
dic36	Japan: Honshu, Miyagi, Sendai, Tohoku University	*Coptis japonica*							LC669884	LC670185	LC670230	Soichi Osozawa
mono38	Japan: Honshu, Miyagi, Sendai, Tohoku University	*Trillium smallii*							LC669885	LC670186	LC670231	Soichi Osozawa
mono40	Japan: Honshu, Miyagi, Sendai, Tohoku University	*Cypripedium japonicum*							LC669886	LC670187	LC670232	Soichi Osozawa
dic42	Japan: Honshu, Miyagi, Sendai, Tohoku University	*Epimedium grandiflorum*							LC669887	LC670188	LC670233	Soichi Osozawa
dic44	Japan: Honshu, Miyagi, Sendai	*Cornus florida*							LC669888	LC670189	LC670234	Soichi Osozawa
dic72	Japan: Honshu, Miyagi, Sendai, Tohoku University	*Gentiana scabra*							LC669889	LC670190	LC670235	Soichi Osozawa
dic80	Japan: Honshu, Miyagi, Kurihara	*Ranunculus nipponicus*							LC669890	LC670191	LC670236	Soichi Osozawa
dic81	Japan: Honshu Tokyo, Tokyo University (New Caledonia origin)	*Amborella trichopoda*							LC669891	LC670192	LC670237	Soichi Osozawa
mono4	Japan: Ryukyu, Tokuno‐shima; Mt. Inokawa‐dake; 645 m	*Arisaema kawashimae*							LC669892	LC670193	LC670238	Soichi Osozawa
mono5	Japan: Honshu, Miyagi, Sendai, Tohoku University	*Iris gracilipes*							LC669893	LC670194	LC670239	Soichi Osozawa
suzu6	Japan: Ryukyu, Okinawa‐jima, Kunigami; Mt. Yonaha‐dake	*Strobilanthes tashiroi*							LC669894	LC670195	LC670240	Soichi Osozawa
suzu8	Japan: Ryukyu, Ishigaki‐jima, Yonehara	*Strobilanthes formosana*							LC669895	LC670196	LC670241	Soichi Osozawa
vio2	Japan: Ryukyu, Amami Oshima	*Viola amamiana*							LC669896	LC670197	LC670242	Kinichi Watanabe
vio3	Japan: Iriomote‐jima; Kanpira waterfall; 100 m	*Viola tashiroi*							LC669897	LC670198	LC670243	Soichi Osozawa
vio4	Japan: Ryukyu, Okinawa‐jima, Onna, Manzamo	*Viola okinawensis*							LC669898	LC670199	LC670244	Tokumasa Shimabukuro
vio5	Japan: Izu, Hachijo‐jima	*Viola grypoceras*							LC669899	LC670200	LC670245	Soichi Osozawa
vio7	Japan: Ryukyu, Kikai‐jima	*Viola betonicifolia*							LC669900	LC670201	LC670246	Soichi Osozawa
vio8	Japan: Ryukyu, Amami Oshima, Tatsugo	*Viola yedoensis*							LC669901	LC670202	LC670247	Soichi Osozawa
vio9	Japan: Ryukyu, Tokuno‐shima, Isen	*Viola yedoensis*							LC669902	LC670203	LC670248	Soichi Osozawa
vio10	Japan: Ryukyu, Tokuno‐shima, Isen	*Viola betonicifolia*							LC669903	LC670204	LC670249	Soichi Osozawa
vio11	Japan: Ryukyu, Okinawa‐jima, Motobu; Mt. Katsuu‐dake	*Viola yedoensis*							LC669904	LC670205	LC670250	Soichi Osozawa
vio14	Taiwan: Yangmingshan	*Viola yedoensis*							LC669905	LC670206	LC670251	Soichi Osozawa
vio15	China: Hong Kong	*Viola* sp.							LC669906	LC670207	LC670252	Soichi Osozawa
vio16	Japan: Ryukyu, Tokara Nakano‐shjima; Mt. O‐take	*Viola grypoceras*							LC669907	LC670208	LC670253	Soichi Osozawa
vio18	Japan: Ryukyu, Kume	*Viola yedoensis*							LC669908	LC670209	LC670254	Soichi Osozawa
vio19	Taiwan: Mt. Hehuan; 3422 m	*Viola* sp.							LC669909	LC670210	LC670255	Soichi Osozawa

Based on the flower structure of *Heterotropa*, it is likely that their pollinators are small flies and millipedes (Hiura 1978), while ants may carry/transport the seeds (Maeda 2013). Consequently, these species have limited dispersal ability (Sugawara 2006), which may contribute to the high proportion of endemic *Heterotropa* species (Kelly 1998; Matsuda et al. 2017; Takahashi and Setoguchi 2017).

*Heterotropa* species exhibit a preference for cool and snowy climates with humidity, commonly found as members of spring ephemerals in beech forests. In the subtropical Ryukyu Islands, their habitat is typically situated near the summits of high mountains, such as Mt. Omoto‐dake on Ishigaki‐jima and Komi‐dake and Goza‐dake on Iriomote‐jima, often shrouded in clouds and cooler compared to the lower elevations (covered by *Pleioblastus linearis*; also the habitat of the endemic butterfly *Ochlodes asahinai*; Figure 1). They are seldom found in humid and relatively cool stream valleys, even within evergreen forests at lower elevations, which could be attributed to seed dispersal toward the base by rainfall (Maeda 2013). In Taiwan, *Heterotropa* species are also restricted to relatively high mountainous areas, including Mt. Datun‐shan with its glassy summit, but also inhabiting cool stream valleys. Note that the evergreen oak trees experience defoliation and leafing in early spring, allowing the *Heterotropa* layer to be exposed to sunlight during the renewal season.

The limitation of their habitat to summit areas on the southern islands suggests that the populations of Ryukyu–Taiwan *Heterotropa* may have been influenced by isolation from one another, even within an island if it contains multiple summits. As a typical example, Tokuno‐shima in the Ryukyu Islands is known to harbor three endemic *Heterotropa* species, and our observations indicate that each endemic species is confined near the summit of three separate high mountains, while the other species, *A. lutchuense*, is found on a lower hillside (fig. 1c in Matsuda et al. 2017).

The formation of the Ryukyu Islands, which began around 1.55 Ma, involved both the subsidence of the islands themselves and the creation of seaways that separated them from the mainland and from each other. At 1.55 Ma (Osozawa et al. 2012), Tokuno‐shima was considerably smaller than its present size, and the three smaller islands were formed with each summit separated by transgressed seaways. Therefore, it is expected that vicariant speciation occurred to give rise to the three endemic species even within Tokuno‐shima. It should be noted, however, that no significant land bridge existed since 1.55 Ma.

Nevertheless, our current study reveals that the vicariance among species on the three isolated summits of Tokuno‐shima is relatively mild or negligible. On the other hand, the species *A. lutchuense*, which occupies lower‐elevation hillsides, belongs to a different subclade and does not share a sister relationship with these three species (cf., Alpine plants in Boucher, Zimmermann, and Conti 2015; the Hawaiian Silversword Alliance in Blonder et al. 2015). Furthermore, while allopatric speciation is expected to have influenced the allopatric island populations of *Heterotropa*, endemic species are not always allopatric and, in some cases, exhibit sympatric distribution within an island, such as in the Amami Oshima islands (Maeda 2013; see fig. 1b in Matsuda et al. 2017). Amami Oshima harbors numerous *Heterotropa* species, and in our analysis, we examined six species. It is possible that not all of these species were solely generated through vicariance events but also involved adaptive radiation processes.

### 2.2 *Asarum* Exact Sampling

*Asarum* species, primarily those from section *Heterotropa*, were collected from Japan, the Ryukyu Islands, and Taiwan (Figures 1, 2, 3, 4, 5; Table 1), with one specimen obtained from Korea. Our collection includes nearly all known *Asarum* species from the Ryukyu Islands, most of which are also listed in table 1 of Takahashi and Setoguchi (2017). Representative *Asarum* calyxes, shown in Figure 4 after being decolorized with ethanol, are provided for reference. Additional details are provided below;

Honshu (highlighted in dark blue in Figure 3): Honshu is the largest island in the Japanese archipelago, and the distribution of section *Heterotropa* species has a northern limit within Honshu. In northern Honshu, *A. megacalyx* is found along the Japan Sea coast, where heavy snowfall occurs (Figure 3). *A. megacalyx* has a large cylindrical calyx (Figure 4) with a dark purplish color. The other species from northern Honshu have a lighter purplish calyx, and species such as *A. fauriei* (the northernmost species of section *Heterotropa*) and *A. ikegamii* are also distributed in areas with heavy snowfall. On the Japan Sea side of central Honshu, *A. megacalyx* is replaced by *A. takaoi*, but *A. takaoi* is also found on the Pacific Ocean coast of the Kii Peninsula. We collected *A. asperum* from Shikoku (highlighted in blue in Figure 3), but this species is also distributed along the Japan Sea coast of western Honshu. *A. sakawanum* is another endemic species found in Shikoku. The *Heterotropa* species in Honshu serve as food plants for *Luehdorfia japonica*, which is exclusively found on Honshu Island.

*Asarum muramatsui*, *A. savatieri*, *A. nipponicum* , and *A. blumei* are distributed in the Southern Fossa Magna, which includes the Izu Peninsula (Osozawa, Ito, et al. 2021). Additionally, *A. tamaense* is found in the Tama Hills, southwest of Tokyo (Figure 3; sequence data sourced from GenBank/DDBJ). The Izu Peninsula, originally located at the northern end of the Izu–Bonin volcanic arc, began to collide and connect with Honshu Island during the Chibanian stage (since 0.77 Ma; Osozawa, Ito, et al. 2021). The Tama Hills, which were submerged by submarine strata until 1.55 Ma (Shinohara et al. 2005), were uplifted and connected with the northwestern Kanto Mountains. These geological changes play a role in understanding the origin of the endemic *Heterotropa* species.

Kyushu and its islets (highlighted in green in Figure 3): There are five endemic species restricted to the main Kyushu Island. These include *A. asaroide* (found in northern Kyushu, but extending to the western end of Honshu), *A. unzen* and *A. kiusianum* (found in northern Kyushu), and *A. hiugana*, *A. satsumense*, and *A. minamitanianum* (restricted to southern Kyushu). The calyx of these species is dark purplish, large, wavy, and balloon or vase shaped, in contrast to the *Heterotropa* species found in Honshu. *A. minamitanianum* has three sepals that extend extremely. *A. crassum* has morphologically similar bell‐shaped calyx to *A. asperum* in Shikoku (highlighted in blue), and it was collected from the Kuro‐shima islet in southwestern Kyushu. The following species have dark purplish and wavy calyxes. Tanega‐shima in the Osumi Islands is home to *A. kumageanum*, while Yaku‐shima (Miyanoura‐dake, 1936 m elevation) has *A. kumageanum* in lowland areas and *A. yakusimense* in highland areas. Nakano‐shima, part of the Tokara volcanic islands, harbors *A. tokarens* and *Viola grypoceras* (see below), which are restricted to the older dissected volcano on the island, while these species are absent on the active Otake volcano at 979 m elevation.

Amami Oshima (highlighted in red in Figure 3): The calyx of *Asarum* species found on Amami Oshima is light purplish and cylindrical. *A. trinacriforme* is distributed in the western half of Amami Oshima, including Uke‐jima islet, while *A. lutchuense* is restricted to the eastern half (Maeda 2013) and their habitats are allopatric. Additionally, we collected *A. fudsinoi* from Mt. Yuwan‐dake (694 m elevation) on the main island of Amami. *A. fudsinoi*, along with similar species such as *A. celsum* (found in the central main island), *A. pellucidum* (Mt. Torigamine, 467 m), and *A. gusk* (found in the central main island), are distributed on the main island. *A. fudsinoi* and these related species are sympatric with *A. lutchuense* (Mt. Yuwan‐dake; these two species, along with *A. lutchuense*, sympatrically occur; Mt. Torigamine; four species sympatrically occur; see detailed fig. 1b in Matsuda et al. 2017).

Tokuno‐shima (highlighted in reddish‐brown in Figure 3) is an island that yields three species of similar endemic *Heterotropa* from three summits: *A. simile* on southern Inokawa‐dake (645 m), *A. hatsushimae* on northern Amagi‐dake (533 m), and *A. leucosepalum* on Sasontsuji‐dake (496 m), just south of Amagi‐dake. Only the latter has a white flower (calyx) and is popularly known as “the first snow” on this subtropical island. The calyx of these three species is distinct from that of another *A. lutchuense* on Tokuno‐shima (the same species found on Amami Oshima). We collected *A. lutchuense* at an altitude of 400 m on a hillside of Sasontsuji‐dake, which is likely its only habitat on Tokuno‐shima, contrasting with Amami Oshima (see fig. 1c in Matsuda et al. 2017).

*Heterotropa* on Okinawa‐jima (highlighted in red in Figure 3) is restricted to the Permian limestone mountains of Katsuu‐dake (452 m) and Yae‐dake (453 m) on the Motobu Peninsula and is not found on summits of the main island such as Yonaha‐dake (Cretaceous pelitic schist; 503 m) covered by *Pleioblastus linearis* (Schoonover and Osozawa 2004; Osozawa and Watanabe 2011). We collected *A. okinawensis* (belonging to section *Heterotropa*) and *A. caudigerum* (belonging to section Euasarum; the only *Asarum* s.s. species known from the Ryukyu Islands), but both have been removed due to human gardening activities.

Yaeyama–Senkaku islands (highlighted in light orange in Figure 3): The calyx of *Asarum* species found in this region is light purplish and cylindrical. *A. dissitum* was collected from the summit of Miyara‐dake (526 m) on Ishigaki‐jima, with one specimen found at the base of this mountain (approximately 300 m). *A. dissitum* is also known from Komi‐dake (469 m) and Goza‐dake (420 m) on Iriomote‐jima, but it was not collected (data from GenBank/DDBJ). Uotsuri‐jima, one of the Senkaku islands located on the northwestern margin of the Okinawa Trough (opposite side of the Ryukyu Islands; Figure 3), yields *A. senkakuinsulare*. A transplanted sample was collected.

Taiwan and Iriomote‐jima (highlighted in orange in Figure 3): The calyx of *Asarum* species found in this region is dark purplish, large, and vase shaped. On Iriomote‐jima Island, we collected *A. gelasinum* from the east (approximately 220 m) of Goza‐dake, and *A. monodoriflorum* at the Kanpira waterfall (100 m) along the Urauchi‐gawa River. Another endemic species, *A. yaeyamensis* , is found in a restricted area, and a vegetated sample was collected. *A. yaeyamensis* was also found in Taiwan (Lu, Chen, and Wang 2009). From northern Taiwan, we collected *A. taitonense* from Datun‐shan volcano (1087 m), and *A. macranthum* from Wulai valley (500 m). The latter species was also found on Alishan (900 m) in southern Taiwan.

The section of Chinese *Heterotropa* was named “section *Longistylis*” by Sinn, Kelly, and Freudenstein (2015b). Sequence data of *Asarum* species belonging to section *Heterotropa* and restricted to southern China (highlighted in Figures 3 and 5) are available from the GenBank/DDBJ. The calyx of these species is dark purplish, but the calyx of *A. forbesii* is light purplish and cylindrical, similar to that of *Asarum takaoi* found in central Honshu.

In the section *Asiasarum*: *A. sieboldii* was collected from four isolated localities along and west of the *Luehdorfia* line (= heavy snow line highlighted in Figure 3; referred to as *A. tohokuense* by Yamaji, Nakamura, et al. 2007) in northern Honshu, where it coexists with *A. megacalyx*. However, *A. sieboldii* is also distributed east of the *Luehdorfia* line, where *A. megacalyx* is replaced by *A. sieboldii* (referred to as *A. tohokuense*). *A. sieboldii* is also distributed in southern Honshu, and *A. dimidiatum* is distributed in the Kii Peninsula, Shikoku, and northern Kyushu (highlighted in Figure 3). We also collected *A. sieboldii* from Busan, Korea (highlighted in Figure 3). *A. sieboldii* is also distributed in southern China (data obtained from GenBank/DDBJ; highlighted in Figure 3), particularly in Hunan, where it is found at hilltops in contrast to *A. forbesii* , which is found at foothills (Li et al. 2011).

In the section *Euasarum*: Apart from the collected *A. caudigerum* from Okinawa‐jima, sequence data, mostly including the Appalachian species, are available from GenBank/DDBJ. We could not collect *A. caulescens* in Japan, and the sequence data for this species are not available (only available for *A. caulescens* from China). Data of section *Hexastylis* from the Appalachian Mountains are also available from GenBank/DDBJ.

Data of *A. yunnanense* from section *Geotaenium* are available from GenBank/DDBJ. We analyzed *Saruma henryi* from the vegetated sample, which is endemic to the Qinling Mountains (Shaanxi), located on the geological border of northern and southern China. *S. henryi* is a spring ephemeral in the Qinling Mountains (Matsumura et al. 2005).

The collected leaf samples used for the present analyses were stored in a desiccator with silica gel filling the space under the platform. Specimen numbers (isolates), species names of *Asarum*, localities, and collectors are registered in the DDBJ/GenBank. The accession numbers, along with specimen numbers (isolates), species names, locality names (countries), and collectors, are listed in Table 1. Voucher specimens were also prepared.

### 2.3 *Viola* and *Fagus* Sampling

*Viola exhibits* its centers of taxonomic and morphological diversity primarily in Mediterranean Europe, Eastern Asia, and North America (refer to fig. 1 in Marcussen et al. 2022). During our collection efforts, we targeted and obtained endemic *Viola* species from the Ryukyu Islands, including *Viola yedoensis*, *V. betonicifolia*, and *V. grypoceras* (Table 1; Figure 1). Data regarding Japanese *Viola* species were sourced from GenBank/DDBJ.

Data on other spring ephemerals such as *Trillium*, *Anemone*, and *Adonis*, as well as *Dioscorea* and *Lilium*, were also obtained from GenBank/DDBJ (Figure 1).

*Fagus* also showcases its centers of diversity in Mediterranean Europe, Eastern Asia, and North America (refer to fig. 4 in Jiang et al. 2021; Cardoni et al. 2022; Momohara and Ito 2023). *Fagus crenata* thrives as a component of beech forests situated on and west of the heavy snow line in northern Honshu (Figures 1 and 3), while *F. japonica* thrives as a component of deciduous forests in regions experiencing a dry climate during winter (where the seasonal western wind is dried by snowfall) along the Pacific side, extending from the backbone range (Figure 3; see details in Worth et al. 2021; partly sympatric). The data for *Fagus* species are sourced from GenBank/DDBJ.

*Quercus* species are prevalent in deciduous forests across Mediterranean Europe, Eastern Asia, and North America (Okaura et al. 2007). *Quercus crispula* is a component of *Fagus crenata* forests, and we additionally included two endemic deciduous oak species, *Q. dentata* and *Q. serrata* , from Japan. Evergreen *Quercus* species are found in the evergreen forests of southern Honshu, as well as the Ryukyu Islands, Taiwan, and southern China. The *Quercus* data are sourced from GenBank/DDBJ.

*Camellia rusticana*, commonly known as snow camellia, thrives in locations with accumulated snow within the *Fagus crenata* forests, while *C. japonica* is typically found in the understory of deciduous forests along the hills of the Pacific coast. The *Camellia* data are sourced from GenBank/DDBJ.

### 2.4 DNA Extraction and Polymerase Chain Reaction Amplification

When applying molecular phylogenetic methods to plants, it was crucial for primers to amplify sequence data that exhibit discernible variation (Kanno et al. 2004; Fazekas et al. 2008), comparable to the variability seen in nuclear genes of animals (Osozawa, Takáhashi, and Wakabayashi 2017a, 2017b; Osozawa, Shiyake, et al. 2017; mitochondrial gene offers greater resolution for phylogenetic analyses). To achieve this, we chose to focus on two specific genes: the chloroplastic maturase K (matK) gene and the ribulose‐1,5‐bisphosphate carboxylase/oxygenase large subunit (rbcL) gene (e.g., the CBOL Plant Working Group 2009), although the rbcL gene alone (see below) does not provide sufficient resolution for species‐level diversification. Additionally, our analyses incorporated the nuclear ribosomal internal transcribed spacer (ITS) region, which has been extensively studied (Kelly 1998; Sugawara et al. 2005; Yamaji, Fukuda, et al. 2007; China Plant BOL Group 2011; Sinn, Kelly, and Freudenstein 2015a, 2015b; Matsuda et al. 2017; Takahashi and Setoguchi 2017; Okuyama et al. 2020) and is known for its ability to discriminate between species.

PopART version 1.7 (Population Analysis with Reticulate Trees; Leigh and Bryant 2015) is now available for analyzing diversification by applying relatively short base pair sequences with lower variance, such as ITS and matK (see detailed application in Osozawa and Nel 2024). These results can then be compared and cross‐checked with the dated tree presented below.

For our analyses, we employed a combination of genes rather than using a concatenated data set and conducted our investigations using BEAST v1.10.4 (Bayesian Evolutionary Analysis Sampling Trees; Suchard et al. 2018). This approach significantly improved resolution, demonstrating the effectiveness of the combined gene analysis strategy. For further details, particularly regarding the relatively short DNA sequences effectively applicable to BEAST v1.10.4, refer to Osozawa (2023) and Osozawa and Nel (2024).

DNA extraction was done using the GenElute Mammalian Genomic DNA Miniprep Kit by Sigma‐Aldrich. Before this operation, crushed young leaf specimens were cleaned with 1% Tris and EDTA solution in 1.5‐mL tube.

Primers used for amplification of the nuclear ribosomal ITS were ITS5F (5‐ GGA AGG AGA AGT CGT AAC AAG G‐3) and ITS4R (5‐ TCC TCC GCT TAT TGA TAT GC ‐3), following the China Plant BOL Group (2011).

Primers used for amplification of chloroplastic matK were matKF (5‐ CGT ACA GTA CTT TTG TGT TTA CGA G‐3) and matKR (5‐ ACC CAG TCC ATC TGG AAA TCT TGG TTC‐3), following the CBOL Plant Working Group (2009). Note that matKF was actually the downstream primer, and matKR was actually the upstream primer.

Primers used for amplification of chloroplastic rbcL were rbcLF (5‐ ATG TCA CCA CAA ACA GAG ACT AAA GC‐3) and rbcLR (5‐ GTA AAA TCA AGT CCA CCR CG‐3), following the CBOL Plant Working Group (2009).

Amplification was done by GoTaq G2 Green Master Mix, Promega, and the temperature of incubation was 94°C for 60 s, with denaturation at 94°C for 30 s, annealing at 53°C for ITS (56°C for matK; 54°C for rbcL) for 60 s, extension at 72°C for 60 s, cycled 35 times, and final extension was at 72°C for 5 min.

The PCR product was purified using Wizard SV Gel and PCR Clean‐Up System, Promega. Sequencing was done by Macrogen Japan.

Sequence alignment was done using ClustalW incorporated in MEGA 11 (Tamura, Stecher, and Kumar 2021). The ITS gene (694 bp), the chloroplastic matK gene (833 bp), and the chloroplastic rbcL gene (544 bp) were readable. Codon translation for the matK and rbcL sequences was checked using the ExPASy‐Translate tool and Bioinformatics Resource Tool. These sequences were also checked by BLAST (Basic Local Alignment Search Tool) offered by DDBJ, and such data are reflected in our registered data in the DDBJ/GenBank (Table 1).

### 2.5 Haplotype Network Analyses

**FIGURE 6:**
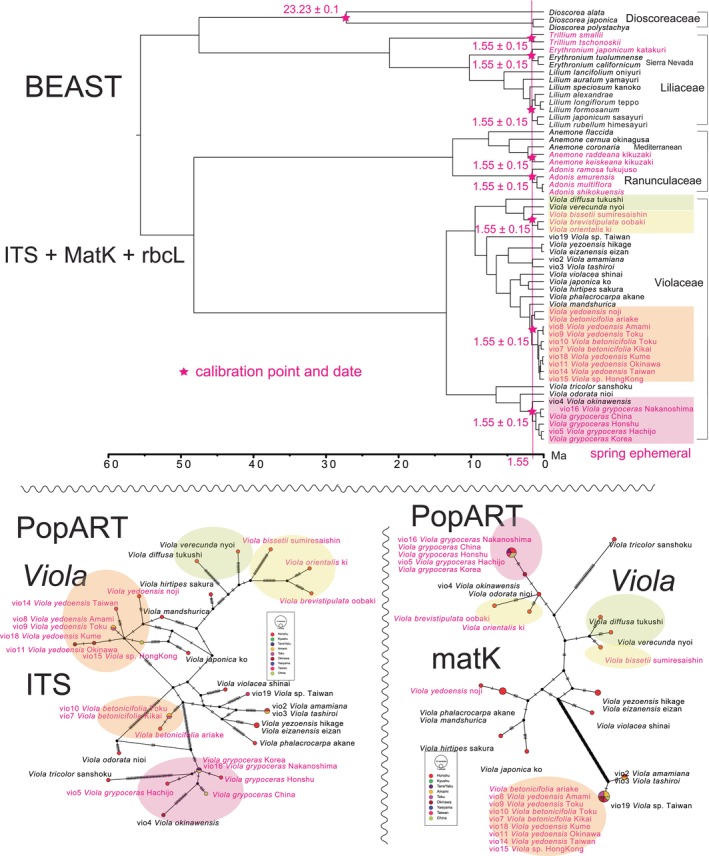
Top: Bayesian inference tree of *Viola* and other spring ephemerals using the combined sequence of ITS (694 bp), matK (833 bp), and rbcL (544 bp) constructed using BEAST v1.10.4. Bottom: ITS and matK haplotype network of *Viola* generated using PopART.

The present analysis is applicable to single‐species variation but can also be extended to interspecies relationships. This is due to the lower genomic variation, which is partly attributed to the limited resolution of ITS and matK‐rbcL markers, as well as the shallower differentiation observed in the BEAST tree for Quaternary‐calibrated clades (Figures 6 top and 7 top).

**FIGURE 7:**
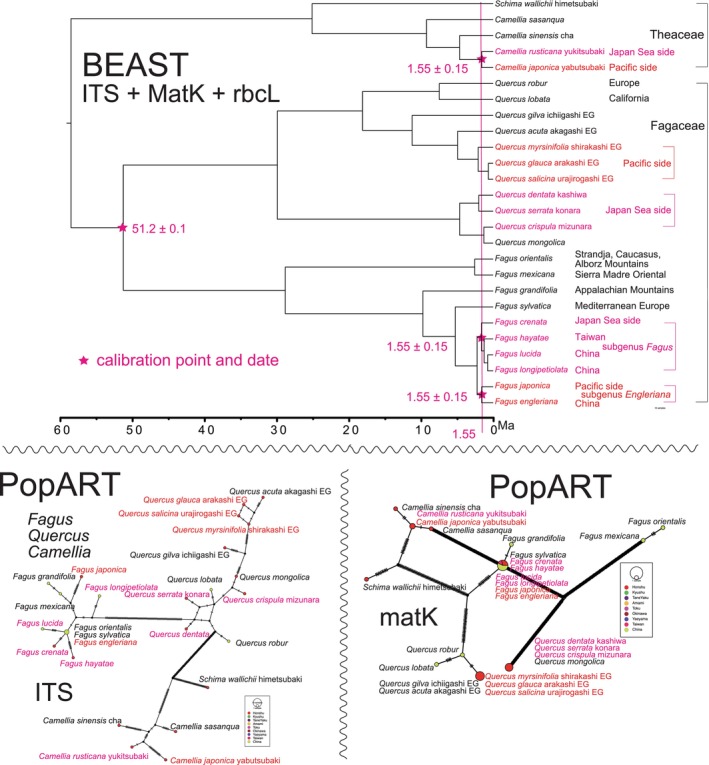
Top: Bayesian inference tree of *Fagus*, *Quercus*, and *Camellia* using the combined sequence of ITS (694 bp), matK (833 bp), and rbcL (544 bp) constructed using BEAST v1.10.4. Bottom: ITS and matK haplotype network of *Fagus–Quercus–Camellia* generated using PopART.

To explore the relationships among ITS and matK haplotypes for *Asarun* (Figure 5 bottom), *Viola* (Figure 6 bottom), and *Fagus–Quercus–Camellia* (Figure 7 bottom), we employed the TCS network (Clement, Posada, and Crandall 2000) in PopART version 1.7 (Population Analysis with Reticulate Trees; Leigh and Bryant 2015). Note that the rbcL haplotype network lacks sufficient resolution to be presented as a figure. For more details, refer to Osozawa and Nel (2024).

### 2.6 Phylogenetic Analyses and Calibration by BEAST

A recent trend involves using voluminous genomic data to construct dated trees (e.g., Okuyama et al. 2020; Tsunenari et al. 2024; Zuntini et al. 2024). However, the present analysis, which combines ITS and matK (plus rbcL), can enhance resolution through robust calibration of the most suitable application (refer to Osozawa 2023; Osozawa and Nel 2024).

This paper presents a newly dated phylogeny of spring ephemerals, including *Asarum* (wild gingers; Figure 5), *Viola* (Figure 6), and *Fagus–Quercus–Camellia* (Figure 7), and crosschecks this phylogeny with a haplotype network constructed using PopART (Figures 5, 6, 7). Previous studies of this nature in the island region of East Asia were lacking, possibly due to the complexity of the evolutionary processes involved, which cannot be easily explained by simple dispersion and colonization models (cf. *Ophiorrhiza japonica*, Nakamura et al. 2010; *Tricyrtis formosana*, Tsunenari et al. 2024; *Asarum*, Takahashi and Setoguchi 2017, Okuyama et al. 2020). Additionally, previous geological models used or assumed in evolutionary studies (e.g., land bridge, Ota 1998) lacked robust geological data, necessitating the development of a new, more rigorous geological model (Osozawa et al. 2012; new data have been incorporated, and a revised version is currently in progress). In a recent study, we examined multiple insect species, revealing compelling indications of vicariance and radiation events linked to the creation of the Ryukyu Islands. The geological event calibration date employed for this analysis was set at 1.55 Ma, as established by previous works (Osozawa et al. 2012, 2013; Osozawa, Takáhashi, and Wakabayashi 2015; Osozawa et al. 2015; Osozawa, Fukuda, et al. 2016; Osozawa, Takáhashi, and Wakabayashi 2017a, 2017b; Osozawa, Shiyake, et al. 2017; Osozawa, Kanai, et al. 2021; Osozawa and Wakabayashi 2015, 2022, latest version 2024). Importantly, it is noteworthy that these publications have effectively circumvented circular reasoning, ensuring the robustness of our findings. Also refer to Osozawa (2023) and Osozawa and Nel (2024).

A Bayesian inference (BI) tree (Figures 5, 6, 7) was constructed using the software BEAST v1.10.4. The following sequence of operations was employed: BEAUti, BEAST, TreeAnnotator, and FigTree. It is necessary to download the BEAGLE Library in advance to utilize the BEAST platform software. For further details, refer to Osozawa (2023) and Osozawa and Nel (2024).

Based on geological evidence for island separation presented by Osozawa et al. (2012), we assumed that the most recent common ancestors (MRCAs) of the ingroup species began differentiating at 1.55 ± 0.15 Ma (Figures 5, 6, 7). To specify the time to the MRCAs for these ingroup species, a date of 1.55 ± 0.15 Ma was input into BEAUti. For further details, refer to Osozawa (2023) and Osozawa and Nel (2024).

Furthermore, fossil data were used for calibration in Figure 5. Fossil leaves of *Piper* (Piperales, paleodicots) were reported from the Maastrichtian coal seams in Colombia, South America (Martinez et al. 2015). Therefore, the time of MRCA of all species of Piperales, including *Piper*, *Houttuynia*, *Aristolochia*, *Saruma*, and *Asarum*, was estimated to be 69.05 ± 3.05 Ma (Figure 5). An Eocene *Aristolochia* fossil was reported from the Green River Formation (Grande 1984), and its Ar‐Ar age has been determined (Smith et al. 2003). Hence, the time of MRCA for *Aristolochia*, *Saruma*, and *Asarum* was estimated to be 51.25 ± 0.31 Ma (Figure 5). Figure 6 was also calibrated using a *Dioscorea* fossil found in Ethiopian strata, which yielded a U–Pb age of 23.23 ± 0.1 Ma (Pan et al. 2014). As compiled by Momohara and Ito (2023), *Fagus langevinii* was reported from the Klondike Mountain Formation in Washington (Manchester and Dillhoff 2004), with a U–Pb age estimated at 51.2 ± 0.1 Ma (Rubino et al. 2021), which was used as the fossil calibration date in Figure 7.

Running BEAST was done by incorporating each xml input file made by BEAUti. The consequent tree was drawn by FigTree v1.4.4, for that, the tree files were input into TreeAnnotator. The BI trees are shown in Figures 5, 6, 7.

## 3 Results

### 3.1 *Asarum* Phylogeny (Figure 5)

In the ITS and matK haplotype networks in Figure 5 bottom for *Asaurum* wild gingers, seven section‐specific clusters are evident: *Saruma henryi*, section *Geotaenium* (refer to Sugarawa, Ogitsu, and Cheng 1990), *Euasarum* (including North America and Europe), *Asiasarum*, *Hexastylis* (North America), *Longistylis*, and core *Heterotropa*. Note that *Asarum forbesii* (*Longistylis*) is included in the core *Heterotropa* cluster in the ITS network and is part of a dominant or root haplotype in the matK network. The ITS clusters are separated by multiple mutation steps, whereas the matK clusters are separated by a single mutation step.

Within the core *Heterotropa* cluster, distinct island or island group clusters are observed, although there are many exceptions. For instance, in the ITS haplotype network, the Honshu cluster contains two Kyushu haplotypes, as34 and as45. The Tane–Yaku–Tokara cluster includes Amami (as16a) and Toku (as12) haplotypes, while the Taiwan haplotypes form a single cluster with Yaeyama (Iriomote) haplotypes. Additionally, the Amami–Toku–Okinawa–Yaeyama (Ishigaki–Iriomote; *Asarum dissitum*) haplotypes constitute a rather composite cluster. However, Amami (as15a) shares a single haplotype with Iriomote (*dissitum*) and Ishigaki (as4b), and Toku (as9) shares a single haplotype with Senkaku (as3). A similar complex cluster pattern is observed in the matK haplotype network for core *Heterotropa*.

In the Aristolochiaceae clade shown in Figure 5 top, the *Saruma henryi* lineage is the most basal after the *Aristolochia* basal clade. The *Asarum* major clade includes section *Geotaenium*, *Euasarum*, *Asiasarum*, *Hexastylis* (North America), *Longistylis*, and core *Heterotropa* clades. Note that only *Asarum forbesii* (*Longistylis*) is included in the core *Heterotropa* clade, and is sister to the clades other than the Honshu (–Shikoku–Kyushu) clade.

In the core *Heterotropa* clade, Figure 5 top reveals significant differentiation into endemic species as relatively cohesive island populations since 1.55 Ma. However, species from the same island do not always form a single clade as might be expected under vicariance scenarios. Instead, they may form different clades, leading to composite species within specific clades that are not composed solely of species from a single island.

Detailed information on the core *Heterotropa* clade is as follows:

In the Honshu (blue)–Shikoku–Kyushu clade, *Heterotropa* species from northern and central Honshu, including the Izu Peninsula and its surroundings, as well as Shikoku and Kyushu, are not monophyletic and are intermingled with each other. *A. megacalyx*, which inhabits areas with heavy snowfall on the Japan seaside of northern Honshu, shows strong differentiation within the species. Four specimens failed PCR amplification and sequencing for ITS, indicating the severe differentiation of this species and primer mismatches. Only *A. megacalyx* has a dark purplish and extremely large calyx.

*A. takaoi* shows a similar DNA sequence among the three analyzed specimens from snowy environments along the Japan Sea side of central Honshu (Figure 3), but it is distinct from the *A. takaoi* population on the Kii Peninsula. *A. takaoi* from central Honshu is a sister species to *A. muramatsui* from the Izu Peninsula. *A. takaoitakaoi* var. *austrokiiense* from the Kii Peninsula is a sister species to *A. savatieri* and *A. nipponicum* from the Southern Fossa Magna. *A. sakawanum* from Shikoku is a sister species to *A. minamitanianum* in southern Kyushu, although the tip of its vase‐shaped calyx is more developed in the latter (Figure 4). *A. asperum* from Shikoku (although this species is mainly found in western Honshu) is a sister species to *A. crassum* on Kuro‐shima islet, located off the southern coast of Kyushu, and they share a similar vase‐shaped calyx form. The sister group relationship of *A. asperum* + *A. crassum* is with *A. blumei* (Southern Fossa Magna) + *A. tamaense* (Tama Hills).

The Tane–Yaku–Tokara (light green)–Amami–Toku clade consists of *A. kumageanum* (found in the lowland of Yaku‐shima and Tanega‐shima, genetically distinct), *A. yakusimense* (Yaku‐shima highland), *A. tokarense* (Nakano‐shima), and *A. lutchuense* (Amami Oshima and Tokuno‐shima). These species are characterized by a wavy and dark purplish calyx.

The Kyushu (dark green) clade includes *A. hiugana*, *A. asaroides*, *A. satsumense*, *A. unzen*, and *A. kiusianum*. These species have a balloon‐shaped and large, dark purplish calyx, particularly notable in *A. asaroides*.

The Amami–Okinawa (red)–Toku clade comprises *A. trinacriforme* (western Amami Oshima), three Tokuno‐shima species from different summits ( *A. simile* , *A. hatsushimae*, and *A. leucosepalum*; the first two species are genetically identical), and *A. okinawense* (Okinawa‐jima). The calyx in this clade is light purplish and cylindrical in shape.

The Amami (red) clade consists of genetically similar species: *A. fudsinoi* (main island), *A. celsum* (central main island), *A. pellucidum* (Mt. Torigamine, 467 m), and *A. gusk* (central main island). These species exhibit a light purplish cylindrical calyx.

The Yaeyama–Senkaku (orange) clade includes *A. dissitum* (found in Ishigaki‐jima and Iriomote‐jima with a sister relationship) and *A. senkakuinsulare* (Senkaku islands). The latter species is not part of the *Longistylis* clade in China, even though the Senkaku islands are located on the margin of the Chinese continental shelf and in a more inward (westward) position relative to the Ryukyu Islands. The calyx in this clade is light purplish and cylindrical.

The Taiwan–Iriomote (reddish orange) clade consists of *A. gelasinum* (genetically identical to *A. monodoriflorum*), *A. yaeyamense* (Iriomote‐jima), *A. taitonense*, and *A. macranthum* (Taiwan). The calyx in this clade is dark purplish, smaller in Iriomote‐jima, and larger in Taiwan.

### 3.2 *Viola* and Spring Ephemeral Phylogeny (Figure 6)

*V. orientalis* and *V. brevistipulata*, both found in the beech forests along the Japanese seaside, diverged around 1.55 Ma. *Viola bissetii* is their sister species. *Viola grypoceras* shows differentiation across various regions, including the main island of Japan, Hachijo‐jima (Izu Islands; cf. Osozawa, Ito, et al. 2021), each island of the Ryukyu Islands, Korea, and China. It is closely related to the endemic species *V. okinawensis*. *V. yedoensis* and its sister species *V. betonicifolia* are sympatrically distributed in Japan and underwent differentiation in the Ryukyu Islands, Taiwan, and China, although their matK sequences are identical.

Endemic species of *Adonis*, *Anemone*, and *Trillium* have emerged since 1.55 Ma (Figure 6 top). *Erythronium japonicum* is differentiated from *E. tuolumnense* and *E. californicum* in the Sierra Nevada. Several species of *Lilium* and *Trillium* are not spring ephemerals but have differentiated around 1.55 Ma.

### 3.3 *Fagus* and Woody Pant Phylogeny (Figure 7)

*Fagus crenata* is a dominant species in the beech forests along the Japanese seaside and is genetically distinct from *Fagus japonica* (cf. Cardoni et al. 2022), which is part of the deciduous forests on the Pacific side. In Figure 7, each *Fagus* species in Japan is a sister to species found in China (including Taiwan), but they form a cluster with Chinese–Taiwanese, European, and North American *Fagus* species.

Deciduous *Quercus* species are important components of the beech forests along the Japanese seaside and underwent differentiation around 1.55 Ma, along with their Chinese counterparts. Several evergreen *Quercus* species, which are major components of the forests on the Pacific side, also differentiated around 1.55 Ma. These evergreen species form a distinct cluster separate from the deciduous *Quercus* species cluster, which includes *Quercus* from California and Europe.

*Camellia rusticana*, found in the beech forests along the Japan seaside, is closely related to *Camellia japonica* , which is prevalent in the evergreen forests on the Pacific side. They underwent differentiation around 1.55 Ma.

## 4 Discussion

### 4.1 Adaptive Radiation Rather Than Allopatric Speciation, Dispersal Origin of Disjunct Distribution, for *Heterotropa*

**FIGURE 8:**
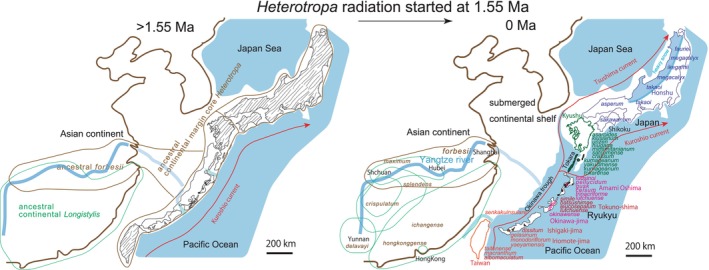
Right figure: Present (0 Ma) habitats of *Heterotropa* in the Japan–Ryukyu–Taiwan islands and China. Colors reflect endemic species from an island or island unit. Note that the same island species are not always allopatric. Left figure: Original (just before 1.55 Ma) habitats of a single ancestral core *Heterotropa* species in the proto‐ Japan–Ryukyu–Taiwan islands (= marginal area of the Asia continent) that of *Asarum forbesii* along the Yangtze River (drowned valley after Ujiie 1998) and those of Chinese *Longistylis*.

*Heterotropa* diversification was partially systematic and region specific, with the primary mechanism being allopatric speciation. However, the mixing of haplotypes or genotypes between regions and the sympatric occurrence within islands suggest another mechanism of adaptive radiation. The extensive radiation scenario is illustrated in Figure 8. The other *Asarum* section including *Longistylis* and North American and European *Euasarum* extensively radiated since 1.55 Ma. Note that Okuyama et al. (2020) estimated the divergence date for core *Heterotropa* at 5 Ma. For more details, including reasons why dispersal is not recommended for *Asarum*, see below.

According to Okuyama et al. (2020), *Asarum forbesii*, the only deciduous *Heterotropa* species native to mainland China, is closely related to the evergreen species of core *Heterotropa* distributed across the Japanese Archipelago and Taiwan. This relationship, similar to ours (Figure 5), suggests that the radiation of core *Heterotropa* may have been facilitated by long‐distance dispersal through the Ryukyu Islands (c.f., Yamaji et al. 2007; Qiu, Fu, and Comes 2011; Takahashi and Setoguchi 2017; Tsunenari et al. 2024). However, we did not find evidence supporting a dispersal event from China to Japan. Instead, we propose that the ancestral habitat of *A. forbesii* extended to the continental margin of the ancestral core Heterotropa (Figure 8 > 1.55 Ma).

Furthermore, according to Okuyama et al. (2020), the core Heterotropa was divided into clades based on representative ingroup species (their fig. 7), which corresponds to the geographically defined clades in our analysis (Figure 5), with the exception of *Asarum okinawense* in Okinawa‐jima (which is an unreasonable inclusion in the Taiwan–Iriomote ingroup in their fig. 7, as opposed to being part of the acceptable Amami–Okinawa–Toku clade in our Figure 5). Our BEAST analysis using traditional Sanger sequence data provides reliable and comparable resolution to the double‐digested RAD (restriction site‐associated DNA) sequence data used by Okuyama et al. (2020); refer to Osozawa 2023, and Osozawa and Nel 2024). Posterior probability on each node is basically 1 or close to 1 in our Figure 5 (not displayed to reduce complexity). Moreover, their study indicates that most estimated dispersal events occurred between adjacent areas over relatively short distances, suggesting that range expansion has been geographically restricted throughout the radiation history of these species.

Due to the connection of the Izu Peninsula with central Honshu after 0.77 Ma and the uplift of the Tama Hills after 1.55 Ma, it is likely that endemic *Heterotropa* species such as *Asarum muramatsui* and *A. tamaense* dispersed from central Honshu. These Izu and marginal Izu species are indeed sister taxa to *A. takaoi* from central Honshu (Figure 5). We support the idea of short‐distance dispersal as a mechanism for radiation. Regarding *A. minamitanianum* (as45) in southern Kyushu and *A. crassum* (as34) in Kuro‐shima islet, south Kyushu, their inclusion in the Honshu clade raises the possibility of dispersal. However, the southwestward dispersal cannot be explained by the reverse directional flow of the Kuroshio current (Figure 3).

In the Tane–Yaku–Tokara + Amami–Toku clade, the genetic distinction between the same species of *A. kumageanum* on Yaku‐shima and Tanega‐shima (as35), as well as *A. lutchuense* on Amami Oshima (as16a) and Tokuno‐shima (as12), suggests that speciation might be related to dispersal driven by the Kuroshio warm current from the south, such as from Tokuno‐shima (Figure 3). Dispersal from Nakano‐shima to Tanega‐shima and Yaku‐shima could be possible through the Kuroshio current. However, long‐distance dispersal from Tokuno‐shima and then toward Yaku‐shima may be challenging due to the presence of the Tokara deep strait (Figure 3; see Osozawa et al. 2012). As for *Asarum okinawense* (as7) on Okinawa‐jima mentioned by Okuyama et al. (2020), it is plausible that dispersal occurred from the Yaeyama Islands through the Kuroshio current, although it would need to cross the Kerama deep strait (Figure 3; see Osozawa et al. 2012). However, this is not supported by our findings (Figures 5 and 8). Furthermore, considering the relatively large seed size, susceptibility to desiccation, presence of ant dispersal syndromes (Okuyama et al. 2020), and the ease with which *Asarum* seeds can dry out when exposed to sunlight (Hiura 1978), it is unlikely that *Asarum* species are capable of migrating across seas.

According to Takahashi and Setoguchi (2017), they proposed that the current species diversity of core *Heterotropa* initially arose through allopatric range fragmentation in East Asia. Hiura (1978) estimated a dispersal rate of 0.5 m/year by combining the rate of seeds carried by ants (5 m/year) with 10 years of flowering since germination, as also suggested by Kelly (1998), Takahashi and Setoguchi (2017), Matsuda et al. (2017), and Okuyama et al. (2020). The formation of the Okinawa Trough since 1.55 Ma resulted in a disjunct distribution (Osozawa et al. 2012), and we propose allopatric and vicariant speciation as the mechanism for generating insect endemic species in Japan, Ryukyu, and Taiwan islands relative to China (Osozawa and Wakabayashi 2015, 2022; Osozawa et al. 2013, Osozawa, Takáhashi, and Wakabayashi 2015; Osozawa et al. 2015; Osozawa, Fukuda, et al. 2016; Osozawa, Takáhashi, and Wakabayashi 2017a, 2017b; Osozawa, Shiyake, et al. 2017; Osozawa, Kanai, et al. 2021; Osozawa, Ogino, et al. 2016). This process may have also acted for *Heterotropa*. Consequently, we calibrated the *Heterotropa* ingroup species (*Longistylis* + core *Heterotropa*) at 1.55 Ma (Figure 5).

The common ancestor of *Heterotropa* initially colonized a wide area extending from south China to Taiwan, Ryukyu, and Japan (Figure 8; note the absence in north China, Korea, and Primorsky), and then started to diverge into geographical clade species of *Longistylis*, *Asarum forbesii*, and core *Heterotropa* at 1.55 Ma (Figure 8). The speciation of *Longistylis* clade species occurred within south China. The core *Heterotropa* consists of geographical clades, each comprising an island or neighboring islands, and a tentative explanation is that the splitting into geographical clade species was the result of vicariance.

In the Kyushu clade, the Beppu–Shimabara graben, a fault‐bounded valley where some faults ruptured during the 2016 Kumamoto earthquake, may act as a physical barrier to migration and dispersal between northern and southern Kyushu, as observed in the distribution of ground beetles (Osozawa, Ogino, et al. 2016). *A. hexalobum* (= *hexaloba*; not collected; analyzed in Okuyama et al. 2020) has two subspecies whose boundaries closely correspond to this barrier (Hiura 1978). However, Okuyama et al. (2020) represented *A. hexalobum* as a sister to the Kyushu clade species in their fig. 6 (in fig. 7, abnormally represented as paraphyletic to all other clades except the Honshu clade). In Figure 5, the Kyushu subclade includes the northern species such as *A. asaroides* (as37) and the southern species such as *A. hiugana* (as32).

In the Amami–Okinawa–Toku clade, Tokuno‐shima was likely lower in elevation around 1.55 Ma compared to the present, based on the distribution of Quaternary marine strata along the island's perimeter. At that time, it is probable that three summits were separated by narrow and shallow seaways. The endemism of *Asarum* on these isolated summits is likely a result of local vicariant speciation that began at 1.55 Ma. However, *A. simile* (as9; southern summit) and *A. hatsushimae* (as10; northern summit) show genetic similarity (similar calyx), while only *A. leucosepalum* (as11; central summit, just south of the northern summit; white coloration of calyx) is distinct (Figure 2).

In the Yaeyama–Senkaku clade, *A. dissitum* in Ishigaki‐jima (as4b, as4c) is a sister to the same species in Iriomote‐jima, and they show mild differentiation within the Yaeyama Islands.

It is important, however, to note that geographical clades do not always consist of pure single island populations like the Kyushu clade, which is the only example of such purity. Instead, they often comprise composite island species (Figure 5; overlapping coloration of each clade of core *Heterotropa*). Alternatively, an island species with sympatric occurrence tends to break up into distinct clades (Figure 5; the same coloration found in distinct clades).

The Kyushu (as45) and Kuro‐shima islet (as34) species belong to the Honshu clade, while the Amami Oshima (as16a) and Tokuno‐shima (as12) species are part of the Tane–Yaku–Tokara (as17) clade. The Amami–Okinawa–Toku clade is distinct from the Amami own clade, and the Yaeyama–Senkaku clade is separate from the Taiwan–Iriomote composite clade. Both Iriomote species are present in the Taiwan–Iriomote clade, while Senkaku species are not found in the *Longistylis* clade.

We propose that in addition to simple vicariance mechanisms, the composite differentiation of the core *Heterotropa* clade, as well as the *Longistylis* clade and *Asarum forbesii*, can be explained by simultaneous genetic and morphological expansion, leading to the generation of numerous endemic species through adaptive radiation from a common ancestor of *Heterotropa* (Figures 5 and 8). Extensive adaptive radiation is evident from the variation in calyx morphology among endemic species and their varieties (Figures 2 and 4). Similar patterns of morphological and genetic differentiation are observed in the Hawaiian Silversword Alliance, supporting the concept of adaptive radiation (Blonder et al. 2015).

Hiura (1978) demonstrated contrasting habitats of *Asarum takaoi* in Quaternary marine strata and *A. asperum* on older metamorphic and igneous rocks in the Kinki district near Osaka, western Honshu. This can be explained by adaptive radiation onto different soil types rather than allopatric speciation.

For the species on Amami Oshima and Tokuno‐shima, Matsuda et al. (2017) suggested that rapid adaptive radiation from a common ancestor occurred over a short period, which may still be incomplete. In the Amami clade (Figure 5), *A. fudsinoi* exhibits various morphotypes of calyx and leaf that resemble distinct species, but genetically it is similar to other *Heterotropa* species in this clade, such as *A. gusk*, *A. celsum*, and *A. pellucidum* , which have distinct morphologies (Figure 2). The presence of *A. trinacriforme* and A. *lutchuense* across multiple clades (Figure 5) can also be explained by adaptive radiation.

In the Taiwan–Iriomote clade, *A. monodoriflorum* (as6) and *A. gelasinumin* (as5b) in Iriomote‐jima are genetically the same (Figure 5). *A. yaeyamense* (as55), found on Iriomote‐jima, was recently identified in northern Taiwan (Lu, Chen, and Wang 2009), and the Yonaguni strait, which is along the route of the Kuroshio current (Figure 3), did not act as an effective barrier between Taiwan and Iriomote‐jima.

Among the analyzed species of section *Asiasarum*, *A. sieboldii* and *A. heterotropoides* show differentiation reflecting their collection sites, although *A. sieboldii* from four collection sites in northern Honshu is genetically the same. However, section *Asiasarum* species do not exhibit allopatry. For example, the habitats of *A. sieboldii* and *A. heterotropoides* in northern Honshu overlap, and sympatric occurrence is particularly observed on the Korean Peninsula (Yamaji, Nakamura, et al. 2007; Yamaji, Fukuda, et al. 2007).

Deciduous species in section *Geotanenium* and its sister of *Euasarum* have stolons, and plant propagation through stolons may be faster than through *Heterotropa* seeds, which are likely dispersed by ants. However, simultaneous radiation at 1.55 Ma is observed, including North American and European species (Figure 5). *A. caudigerum* shows differentiation between Okinawa‐jima (as8a), China, and Taiwan (Figure 5). However, *A. caulescens* is widely distributed in Japan and China, and these *Euasarum* species are sympatrically found.

### 4.2 Synchronous Radiation of Spring Ephemerals

*Viola* and other spring ephemerals have undergone radiation since 1.55 Ma as *Heterotropa*.

*Viola bissetii* and *V. brevistipulata* in the beech forest along the Japan Sea side, *Viola grypoceras* in the deciduous oak forest on the Pacific side, and *Viola yedoensis* and *V. betonicifolia* in the forested areas including the Ryukyu Islands have undergone radiation since 1.55 Ma (Figure 6). Spring ephemerals such as *Adonis*, *Anemone*, *Trillium*, and *Erythronium japonicum* and North American *Erythronium* have also undergone differentiation since 1.55 Ma (Figure 6), becoming key components of these ecosystems.

*Fagus crenata*, which is found in the Japan Sea side beech forest, and *F. japonica* , which is present in the deciduous forest on the Pacific side, have both emerged as a result of the 1.55 Ma radiation (Figure 7; cf., Cardoni et al. 2022). This radiation is also observed in North American and European *Fagus* species. The deciduous *Quercus* species, found in the beech forest on the Japan Sea side, and the evergreen *Quercus* species, found in the Pacific side forest, have also radiated since 1.55 Ma (Figure 7). Additionally, *Camellia rusticana* (snowy camellia) in the Japan Sea side beech forest and *C. japonica* in the evergreen forest on the Pacific side have evolved since 1.55 Ma (Figure 7).

### 4.3 East Asian Spring Ephemerals Were Originated From Quasi‐Local Tectonics‐Induced Quaternary Climatic Change and Adaptive Radiation

Representative species of spring ephemerals include *Viola*, *Adonis*, *Anemone*, *Trillium*, and *Erythronium*, along with *Asarum* found within *Fagus* and deciduous *Quercus* forests. Our research indicates that these species have undergone adaptive radiation since approximately 1.55 Ma. We conclude that the occurrence of these spring ephemerals in northern Japan and East Asia (including North America and Europe) can be attributed, at least in part, to the Quaternary adaptive radiation. Note that spring ephemerals from the Junggar Basin, referred to as desert ephemerals (Qiu et al. 2018; Xiao et al. 2024), are beyond the scope of this study.

One possible trigger for this adaptive radiation could be the environmental changes associated with subsidence and transgression, leading to the formation of isolated islands, which began around 1.55 Ma (Osozawa et al. 2012). These changes likely resulted in significant shifts in climate, including rainfall patterns, as these land masses became surrounded by the sea (see Qiu, Fu, and Comes 2011, for related findings). An illustration of the impact of this event on the region is the notable influence to the strength of typhoons along their paths between the Ryukyu Islands and the mainland (Osozawa and Wakabayashi 2015; Osozawa, Kanai, et al. 2021).

According to data from the Japan Meteorological Agency, the highest annual precipitation in Japan and the Ryukyu region is recorded on Yaku‐shima (4723 mm/year; Miyanoura‐dake, 1936 m). In the Ryukyu region, Amami Oshima (Yuwan‐dake, 694 m) is also a mountainous island with a high annual precipitation of 3054 mm/year, while Iriomote‐jima (Komi‐dake, 469 m) receives an annual precipitation of 2305 mm/year. The high mountains of Taiwan, such as Alishan (2663 m), receive substantial annual precipitation recording 3910 mm/year. These humid islands are characterized by lower‐altitude vegetation and a notable diversity of *Heterotropa* species.

The inflow of the Tsushima warm current into the Japan Sea commenced around 1.55 Ma (Osozawa et al. 2012), leading to significant climate changes in the Japan Sea coastal areas. During winter, the northwest seasonal wind crossing the Japan Sea picks up moisture from the warm water, resulting in heavy snowfall in the coastal areas along the western front of the northern Honshu backbone range, as previously noted. Similar to *Camellia rusticana* and *Fagus crenata*, *A. megacalyx* may have adapted to such a snowy environment and subsequently experienced radiation in this region, also as previously mentioned. Fossil evidence also indicates that the dominance of *F. crenata* coincided with the onset of the inflow of the Tsushima Warm Current into the Japan Sea (Momohara and Ito 2023; cf., Ueki and Tojo 2023).

The establishment of four distinct seasons in the Quaternary is crucial for the survival and development of spring ephemerals, and the timing of their initiation is a significant factor. Evidence suggests that the modern East Asian monsoon began during the late Oligocene, as indicated by stable carbon isotope measurements taken from well‐preserved fossil wood (Vornlocher et al. 2021). Fossil woods from the Miocene period also exhibit clear growth rings, indicating pronounced seasonality during that time (Suzuki 1985; Choi et al. 2010). Notably, a fossil forest consisting of *Metasequoia glyptostroboides* has been discovered in the Pliocene Hirosegawa Tuff (associated with pyroclastic flow deposits; Kitamura et al. 1986), with a U–Pb age estimation of 3.44 Ma (Osozawa and Ito 2023). Artifacts crafted from fossil wood with distinct growth rings can be found in the Aoba‐jo castle, Sendai.

The Quaternary period experienced significant global climatic and environmental changes, including cycles of glaciation and interglaciation, which likely influenced the evolution of East Asian spring ephemerals (cf., Osozawa 2023; Osozawa and Nel 2024). During glacial periods, it is anticipated that there was reduced snowfall and rainfall due to the regression of the Japan Sea area and a decrease in typhoon activity. The adaptive radiation of spring ephemerals in Mediterranean Europe and North America (several Mediterranean European and North American species are included in Figures 5, 6, 7) can also be attributed to the global climatic changes of the Quaternary period.

## 5 Conclusion

The origin of East Asian spring ephemerals can be attributed to the Quaternary adaptive radiation, which was a primary result of tectonically induced climatic changes. These global changes, additionally locally driven by geological processes, played a crucial role in shaping the evolution and distribution of the special ecosystem in East Asia and probably Mediterranean Europe and North America.

## Conflicts of Interest

The authors declare no conflicts of interest.

## Data Availability

All relevant data are within the manuscript. Data in Table 1 are available at GenBank/DDBJ, and accession numbers are in Table 1.
